# Crystal structures of FolM alternative dihydrofolate reductase 1 from *Brucella suis* and *Brucella canis*

**DOI:** 10.1107/S2053230X21013078

**Published:** 2022-01-01

**Authors:** Imani Porter, Trinity Neal, Zion Walker, Dylan Hayes, Kayla Fowler, Nyah Billups, Anais Rhoades, Christian Smith, Kaelyn Smith, Bart L. Staker, David M. Dranow, Stephen J. Mayclin, Sandhya Subramanian, Thomas E. Edwards, Peter J. Myler, Oluwatoyin A. Asojo

**Affiliations:** a Department of Chemistry and Biochemistry, Hampton University, 100 William R. Harvey Way, Hampton, VA 23668, USA; b Seattle Structural Genomics Center for Infectious Disease (SSGCID), Seattle, Washington, USA; c Center for Infectious Disease Research, formerly Seattle Biomedical Research Institute, 307 Westlake Avenue North Suite 500, Seattle, Washington, USA; d Beryllium Discovery, Bainbridge Island, WA 98110, USA

**Keywords:** oxidoreductases, short-chain dehydrogenase/reductase family, dihydrofolate reductases, NADPH, *Brucella suis*, *Brucella canis*, Seattle Structural Genomics Center for Infectious Disease, SSGCID

## Abstract

Members of the bacterial genus *Brucella* cause brucellosis, a zoonotic disease that affects both livestock and wildlife. *Brucella* are category B infectious agents that can be aerosolized for biological warfare. As part of the structural genomics studies at the Seattle Structural Genomics Center for Infectious Disease (SSGCID), FolM alternative dihydrofolate reductases 1 from *Brucella suis* and *Brucella canis* were produced and their structures are reported. The enzymes share ∼95% sequence identity but have less than 33% sequence identity to other homologues with known structure. The structures are prototypical NADPH-dependent short-chain reductases that share their highest tertiary-structural similarity with protozoan pteridine reductases, which are being investigated for rational therapeutic development.

## Abstract 2

Crystal structures of FolM alternative dihydrofolate reductase 1 from *Brucella suis* and *Brucella canis* reveal prototypical NADPH-dependent short-chain reductases with structural similarity to protozoan pteridine reductases that are potential drug targets.

## 1. Introduction

Brucellosis is the most common bacterial zoonotic disease and is caused by the bacterial genus *Brucella*, which infects humans who consume contaminated animal products, or through contact with infected animals and their secretions (Ducrotoy *et al.*, 2016 ▸; Godfroid, Al Dahouk *et al.*, 2013 ▸). *Brucella* are classified as category B infectious agents that can be aerosolized (de Figueiredo *et al.*, 2015 ▸). Serological evidence suggests that human brucellosis is misdiagnosed as malaria or other febrile diseases in sub-Saharan Africa (Ducrotoy *et al.*, 2017 ▸). Brucellosis is highly contagious and affects economically important livestock and wild animals globally (Ducrotoy *et al.*, 2017 ▸; Godfroid, Garin-Bastuji *et al.*, 2013 ▸; Godfroid *et al.*, 2011 ▸; Megersa *et al.*, 2011 ▸). While brucellosis has been eradicated in cattle and small ruminants in a few countries, it remains endemic globally within a wide range of animal hosts (Moreno, 2014 ▸).

Current control approaches for brucellosis include vaccination, education and basic hygiene; however, these strategies have not effectively reduced the disease burden due to cost and other issues (Ariza *et al.*, 2007 ▸). Notably, current vaccines are species-specific and are devastating to pregnant livestock, and cultural practices among rural dwellers and nomadic groups that rear animals are often incompatible with disease control (Ducrotoy *et al.*, 2017 ▸; Godfroid, Al Dahouk *et al.*, 2013 ▸). There is a continued need to develop new cost-effective approaches to treat infected animals, including the rational design or repurposing of small molecules that target enzymes that are vital for bacterial survival. The Seattle Structural Genomics Center for Infectious Disease (SSGCID) has determined the crystal structures of many target enzymes, including FolM alternative dihydrofolate reductase 1 from two *Brucella* species, *B. suis* and *B. canis*. Dihydrofolate reductase reduces dihydrofolic acid to tetrahydrofolic acid using reduced nicotinamide adenine dinucleotide phosphate (NADPH) as the electron donor. While this reaction is catalyzed by the enzyme dihydrofolate reductase (DHFR) in mammals and other organisms, some bacteria have an alternative pathway for reduced folate biosynthesis using FolM alternative di­hydrofolate reductase 1 (Levin *et al.*, 2004 ▸). Here, we present the crystal structures of FolM alternative dihydro­folate reductase 1 from two *Brucella* species, *B. suis* (*Bs*FolM) and *B. canis* (*Bc*FolM).

*Bs*FolM and *Bc*FolM are 95% identical in sequence. *BLAST* alignment of the protein sequences against the Protein Data Bank (PDB) reveals the most similar proteins to be Tt0495 from *Thermus thermophilus* HB8 (Pampa *et al.*, 2014 ▸) with ∼32% sequence identity and ∼85% coverage; *Leishmania major* pteridine reductase (Schüttelkopf *et al.*, 2005 ▸) with ∼30% sequence identity and ∼90% coverage; *Mycobacterium smegmatis* short-chain reductase (Blaise *et al.*, 2017 ▸) with ∼33% sequence identity and ∼85% coverage; and *Trypanosoma cruzi* pteridine reductase 2 (Schormann *et al.*, 2005 ▸) with ∼30% sequence identity and ∼88% coverage. The reported crystal structures of *Bs*FolM and *Bc*FolM are the first steps towards identifying new therapeutics for brucellosis.

## 2. Materials and methods

### 2.1. Macromolecule production

Cloning, expression and purification were conducted as part of the Seattle Structural Genomics Center for Infectious Disease (SSGCID) following standard protocols described previously (Myler *et al.*, 2009 ▸; Stacy *et al.*, 2011 ▸; Bryan *et al.*, 2011 ▸; Choi *et al.*, 2011 ▸; Serbzhinskiy *et al.*, 2015 ▸). The full-length FolM genes from *B. suis* (UniProt A0A0H3G2T6) and *B. canis* (UniProt A9MA73) were PCR-amplified from genomic DNA using the primers shown in Tables 1 ▸ and 2 ▸, respectively. The resultant amplicons were cloned into the ligation-independent cloning (LIC; Aslanidis & de Jong, 1990 ▸) expression vector pBG1861 encoding a noncleavable 6×His fusion tag (MAHHHHHHM-ORF). The plasmids containing A0A0H3G2T6 and A9MA73 were tested for expression and 2 l of culture was grown using auto-induction medium (Studier, 2005 ▸) in a LEX Bioreactor (Epiphyte Three). The expression clones for BrsuA.00010.a.B1.GE36748 and BrcaA.00010.a.B1.GE38297 are available at https://www.ssgcid.org/available-materials/expression-clones/.

His-*Bs*FolM and His-*Bc*FolM were purified in a two-step protocol consisting of an Ni^2+^-affinity chromatography step and size-exclusion chromatography (SEC). All chromatography runs were performed on an ÄKTApurifier 10 (GE) using automated IMAC and SEC programs according to previously described procedures (Bryan *et al.*, 2011 ▸). Thawed bacterial pellets were lysed by sonication in 200 ml lysis buffer [25 m*M* HEPES pH 7.0, 500 m*M* NaCl, 5% glycerol, 0.5% CHAPS, 30 m*M* imidazole, 10 m*M* MgCl_2_, 1 m*M* tris(2-carboxyethyl)phosphine (TCEP), 250 µg ml^−1^ 4-benzenesulfonyl fluoride hydrochloride (AEBSF), 0.025% azide]. After sonication, the crude lysate was clarified with 20 µl (25 units µl^−1^) benzonase and incubated while mixing at room temperature for 45 min. The lysate was then clarified by centrifugation at 10 000 rev min^−1^ for 1 h using a Sorvall centrifuge (Thermo Scientific). In the IMAC step, the clarified supernatant was passed over an Ni–NTA HisTrap FF 5 ml column (GE Healthcare) pre-equilibrated with loading buffer (25 m*M* HEPES pH 7.0, 500 m*M* NaCl, 5% glycerol, 30 m*M* imidazole, 1 m*M* TCEP, 0.025% sodium azide). The column was washed with 20 column volumes (CV) of loading buffer and eluted with a linear gradient over 7 CV of loading buffer plus 250 m*M* imidazole. Peak fractions, as determined by UV at 280 nm, were pooled and concentrated. A SEC column (Superdex 75, GE Healthcare) was equilibrated with running buffer (25 m*M* HEPES pH 7.0, 500 m*M* NaCl, 5% glycerol, 2 m*M* DTT, 0.025% azide). The peak fractions were collected and analyzed by SDS–PAGE. The SEC peak fractions eluted as a single large peak at a molecular mass of ∼77 kDa, suggesting an oligomer, most likely dimeric, trimeric or tetrameric enzyme. The peak fractions were pooled and concentrated to 28.5 mg ml^−1^ (His-*Bs*FolM) or 32.3 mg ml^−1^ (His-*Bc*FolM) as assessed by the OD_280_ using an Amicon concentration system (Millipore). Aliquots of 200 µl were flash-frozen in liquid nitrogen and stored at −80°C until use for crystallization.

### 2.2. Crystallization

Purified His-*Bs*FolM and His-*Bc*FolM were screened for crystallization in 96-well sitting-drop plates against the JCSG++ HTS (Jena Bioscience) and MCSG1 (Molecular Dimensions) crystallization screens. Equal volumes of protein solution (0.4 µl) and precipitant solution were set up at 290 K against 80 µl reservoir in sitting-drop vapor-diffusion format. Before crystallization, NADPH was added to the protein solution to a final concentration of 4 m*M* (*Bs*FolM) or 6 m*M* (*Bc*FolM). The precipitant solution was MCSG-1 condition A1 (Tables 3 ▸ and 4 ▸). The crystals were harvested and cryoprotected with crystallization solution supplemented with 20% ethylene glycol before flash-cooling in liquid nitrogen.

### 2.3. Data collection and processing

Data were collected at 100 K at the Advanced Photon Source, Argonne National Laboratory (Table 5 ▸). The data were reduced with *XSCALE* (Kabsch, 2010 ▸). Raw X-ray diffraction images are available at the Integrated Resource for Reproducibility in Macromolecular Crystallography at https://www.proteindiffraction.org/.

### 2.4. Structure solution and refinement

Both structures were solved by molecular replacement. *Bc*FolM was solved with *BALBES* (Long *et al.*, 2008 ▸) with PDB entry 2uvd, a 3-oxoacyl-(acyl carrier protein) reductase (Ba3989) from *Bacillus anthracis* (Zaccai *et al.*, 2008 ▸), as the search model. *Bs*FolM was solved with *MoRDa* (Vagin & Lebedev, 2015 ▸) using *Bc*FolM (PDB entry 5bt9) as the search model. Both structures were refined using iterative cycles of refinement in *Phenix* (Liebschner *et al.*, 2019 ▸) followed by manual structure-rebuilding cycles in *Coot* (Emsley & Cowtan, 2004 ▸; Emsley *et al.*, 2010 ▸). The quality of both structures was checked using *MolProbity* (Chen *et al.*, 2010 ▸). All data-reduction and refinement statistics are shown in Table 6 ▸. The *Bs*FolM structure was refined to a resolution of 1.70 Å, while that of *Bc*FolM was refined to 1.50 Å resolution. Figures depicting the structure were analyzed and prepared using *PyMOL* (version 1.5; Schrödinger). Multiple sequence alignments were performed using *Clustal Omega* (Li, 2003 ▸; Sievers *et al.*, 2011 ▸). Coordinates and structure factors have been deposited in the Protein Data Bank (https://www.rcsb.org/) as entries 5tgd and 5bt9 for *Bs*FolM and *Bc*FolM, respectively.

## 3. Results and discussion

**Figure 1:**
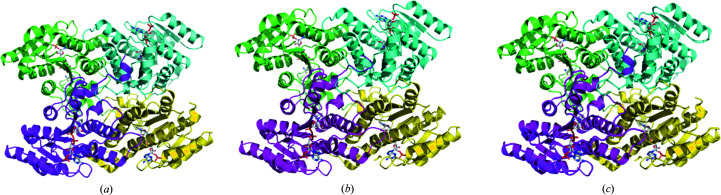
(*a*) *Bs*FolM and (*b*) *Bc*FolM assemble as prototypical FolM alternative dihydrofolate reductase 1 tetramers. (*c*) The *Bs*FolM and *Bc*FolM tetramers are almost identical based on their structural alignment.

The structures of FolM alternative dihydrofolate reductase 1 from *B. suis* (*Bs*FolM) and *B. canis* (*Bc*FolM) were determined in the monoclinic space group *P*2_1_ with four monomers in the asymmetric unit (Fig. 1 ▸). *PDBsum* analysis (http://www.ebi.ac.uk/pdbsum/) indicates that each monomer interacts with three other monomers, with two large interactions and one smaller interaction. The buried surface areas of the inter­actions are ∼1400, ∼1300 and ∼770 Å^2^ per monomer. These surface areas involve 31, 25 and 14 interface amino acids per monomer, respectively. The interface interactions are mostly hydrogen bonds and other nonbonded contacts. The tetramers are similar and superpose with an r.m.s.d. of ∼0.5 Å (Fig. 1 ▸ *c*). The tetramer is the prototypical short-chain dehydrogenase/reductase (SDR) tetramer, suggesting that the single SEC peak may indeed correspond to a tetramer.

Each monomer has the extended double-Rossmann fold of NADPH-dependent SDRs with a central seven-stranded parallel β-sheet sandwiched between two pairs of three α-helices. Both the *Bs*FolM and *Bc*FolM structures were co-crystallized with a cofactor (NADPH). The monomers are virtually identical, with an r.m.s.d. of ∼0.17 Å on superposing all main-chain atoms of both structures (Fig. 1 ▸).

The most similar structures to *Bs*FolM and *Bc*FolM were identified by *PDBeFold* (http://www.ebi.ac.uk/msd-srv/ssm) analysis using the default threshold cutoffs of 70% for the percentage of the secondary structure of the target chain identified in the query protein and of the secondary structure of the query chain (Krissinel & Henrick, 2004 ▸). The most similar structures are protozoan pteridine reductases (Khalaf *et al.*, 2014 ▸; Tulloch *et al.*, 2010 ▸; Schormann *et al.*, 2005 ▸). These structures share ∼29% sequence identity with *Bs*FolM and *Bc*FolM, and their main-chain C^α^ atoms align with an r.m.s.d. of ∼1.5 Å. These protozoan pteridine reductases are more similar to *Bs*FolM and *Bc*FolM than to the structures from *Bacillus anthracis* (Zaccai *et al.*, 2008 ▸), *Streptomyces* (Wang *et al.*, 2014 ▸), *Serratia marcescens* (Liu *et al.*, 2018 ▸), *Thermus thermophilus* (Asada *et al.*, 2009 ▸) or other bacteria.

**Figure 2:**
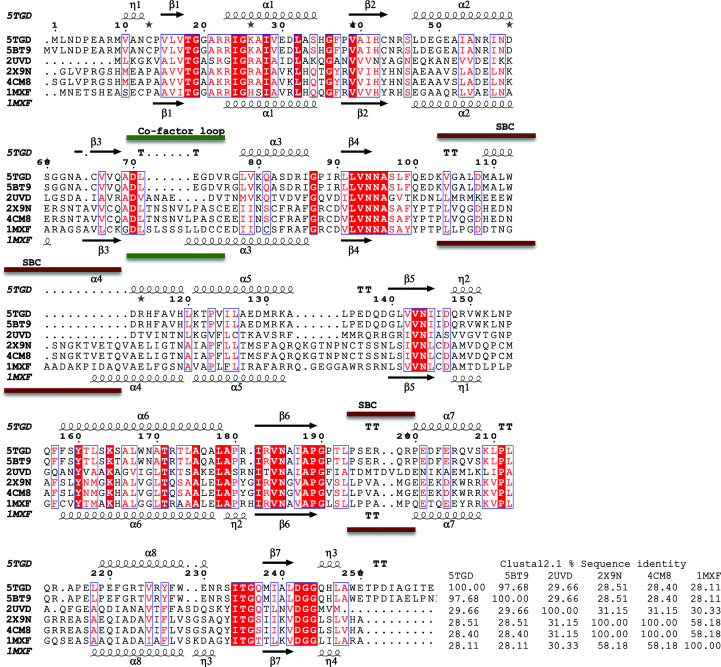
Structural and primary-sequence alignment of FolM alternative dihydrofolate reductase 1 from *B. suis* (PDB entry 5tgd) and *B. canis* (PDB entry 5bt9) with the molecular-replacement search model 3-oxoacyl-(acyl carrier protein) reductase from *Bacillus anthracis* (PDB entry 2uvd) and protozoan structures (*Trypanosoma brucei* pteridine reductase with cyromazine, PDB entry 2x9n; *T. brucei* pteridine reductase ternary complex with cofactor and inhibitor, PDB entry 4cm8; *T. cruzi* pteridine reductase, PDB entry 1mxf). The secondary-structure elements are shown as follows: α-helices are shown as large coils, 3_10_-helices are shown as small coils labeled η, β-strands are shown as arrows labeled β and β-turns are labeled TT. Identical residues are shown on a red background, with conserved residues in red and conserved regions in blue boxes. Regions of greatest variability within the core of the protein are identified with brown lines and labeled SBC due to their proximity to the substrate-binding cavity.

**Figure 3:**
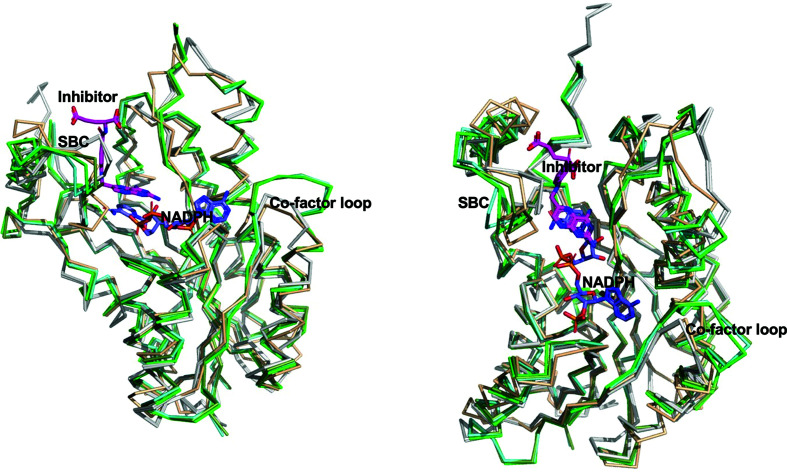
Two views comparing *Bs*FolM and *Bc*FolM monomers with similar structures. The *Bs*FolM and *Bc*FolM monomers (gray) have the prototypical double-Rossmann fold of NADPH-dependent short-chain dehydrogenase/reductases observed in the molecular-replacement search model (tan) and protozoan pteridine reductase (green). The superposed protozoan structures are *Trypanosoma brucei* pteridine reductase with cyromazine (PDB entry 2x9n; cyan green), *T. brucei* pteridine reductase ternary complex with cofactor and inhibitor (PDB entry 4cm8; dark green) and *T. cruzi* pteridine reductase (PDB entry 1mxf; light green). The cofactor NADPH is shown in blue sticks, while the inhibitor from PDB entry 1mxf is shown as magenta sticks in the substrate-binding cavity. As in Fig. 2 ▸, SBC stands for substrate-binding cavity.

The *Bs*FolM and *Bc*FolM structures are in the closed conformation with ordered substrate-binding loops, as observed in protozoan pteridine reductases (Khalaf *et al.*, 2014 ▸; Tulloch *et al.*, 2010 ▸; Schormann *et al.*, 2005 ▸; Schüttelkopf *et al.*, 2005 ▸). Despite being identified as the closest structures by *PDBeFold*, the *Trypanosoma* proteins share a lower sequence identity to *Bs*FolM and *Bc*FolM than the MR search model from *B. anthracis* (Zaccai *et al.*, 2008 ▸), which shares ∼30% sequence identity with both proteins (Fig. 2 ▸). Both structures have structural differences from the molecular-replacement search model, the 3-oxoacyl-(acyl carrier protein) reductase (Ba3989) from *Bacillus anthracis*, and have an r.m.s.d. of ∼2.12 Å on superposing all main-chain atoms (Fig. 3 ▸).

**Figure 4:**
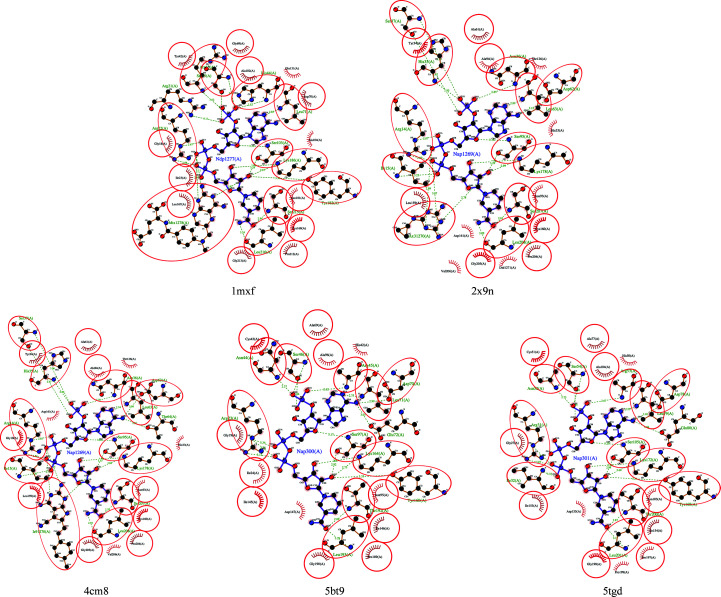
*LIGPLOT* diagrams reveal well conserved NADPH-binding cavities in FolM alternative dihydrofolate reductase 1 from *B. suis* (PDB entry 5tgd) and *B. canis* (PDB entry 5bt9), *Trypanosoma brucei* pteridine reductase with cyromazine (PDB entry 2x9n) *T. brucei* pteridine reductase in a ternary complex with cofactor and inhibitor (PDB entry 4cm8) and *T. cruzi* pteridine reductase (PDB entry 1mxf). Identical amino-acid residues are circled.

While the cofactor-binding cavities of *Bs*FolM, *Bc*FolM and the *Trypanosoma* proteins are well conserved, there is a loop insertion (labeled in green; Fig. 2 ▸). This loop (labeled the cofactor loop in Fig. 3 ▸) points away from the cofactor (NADPH) and aligns well in both *Bs*FolM and *Bc*FolM. Interestingly, this loop is conserved in the protozoan enzymes and forms a 6.5 Å larger cavity than that observed in the *Brucella* enzymes (both *Bs*FolM and *Bc*FolM; Fig. 3 ▸). Apart from this loop region, the cofactor-binding cavity is very similar in these enzymes. Furthermore, the residues involved in NADPH binding are well conserved (Fig. 4 ▸).

As expected, the substrate-binding cavity of each protein shows the greatest structural difference (Figs. 2 ▸ and 3 ▸). This structural variability is believed to allow substrate specificity among SDRs. While the substrates of *Bs*FolM and *Bc*FolM are unknown, their substrate-binding cavities are large enough to accommodate the inhibitors identified by rational therapeutics discovery for human African trypanosomiasis and Chagas disease. There are >150 structures of complexes of protozoan pteridine reductases with unique inhibitors deposited in the Protein Data Bank (Khalaf *et al.*, 2014 ▸; Tulloch *et al.*, 2010 ▸; Schormann *et al.*, 2005 ▸) that can serve as starting points for the discovery of therapeutics for brucellosis.

## 4. Conclusions

The high-resolution structures of FolM alternative dihydro­folate reductase 1 from *B. suis* and *B. canis* have prototypical NADPH-dependent short-chain reductase topology and structural similarity to the well characterized protozoan pteridine reductases. Despite their low sequence identity, their structural similarity to the protozoan pteridine reductases may accelerate drug-repurposing efforts.

## Supplementary Material

PDB reference: *Bs*FolM, 5tgd

PDB reference: *Bc*FolM, 5bt9

## Figures and Tables

**Table 1:** Production of FolM alternative dihydrofolate reductase 1 from *B. suis*

Source organism	*Brucella suis* 1330
DNA source	Dr Jean-Jacques Letesson (University of Namur, Belgium)
Forward primer	5′-CTCACCACCACCACCACCATATGGTGTTGAATGATCCCGAAGC-3′
Reverse primer	5′-ATCCTATCTTACTCACTTATTCGGTAATTCCTGCAATGTCGG-3′
Expression vector	pBG1861
Expression host	*E. coli* BL21(DE3)R3 Rosetta cells
Complete amino-acid sequence of the construct produced	MAHHHHHHMLNDPEARMVANCPVLVTGGARRIGKAIVEDLASHGFPVAIHCNRSLDEGEAIANRINDSGGNACVVQADLEGDVRGLVKQASDRIGPIRLLVNNASLFQEDKVGALDMALWDRHFAVHLKTPVILAEDMRKALPEDQDGLVVNIIDQRVWKLNPQFFSYTLSKSALWNATRTLAQALAPRIRVNAIAPGPTLPSERQRPEDFERQVSKLPLQRAPELPEFGRTVRYFWENRSITGQMIALDGGQHLAWETPDIAGITE

**Table 2:** Production of FolM alternative dihydrofolate reductase 1 from *B. canis*

Source organism	*Brucella canis* RM-666 (NCTC 10854)
DNA source	ATCC 23365
Forward primer	5′-CTCACCACCACCACCACCATATGGTGTTGAATGATCCCGAAGC-3′
Reverse primer	5′-ATCCTATCTTACTCACTTATTCGGTAATTCCTGCAATGTCGG-3′
Expression vector	pBG1861
Expression host	*E. coli* BL21(DE3)R3 Rosetta cells
Complete amino-acid sequence of the construct produced	MAHHHHHHMVLNDPEARMVANCPVLVTGGARRIGKAIVEDLASHGFPVAIHCNRSLDEGEAIANRINDSGGNACVVQADLEGDVRGLVKQASDRIGPIRLLVNNASLFQEDKVGALDMALWDRHFAVHLKTPVILAEDMRKALPEDQDGLVVNIIDQRVWKLNPQFFSYTLSKTALWNATRTLAQALAPRIRVNAIAPGPTLPSERQRPEDFERQVSKLPLQRAPELPEFGRTVRYFWENRSITGQMIALDGGQHLAWETPDIAELPNK

**Table 3:** Crystallization of FolM alternative dihydrofolate reductase 1 from *B. suis* (*Bs*FolM)

Method	Vapor diffusion, sitting drop
Plate type	Rigaku Reagents XJR
Temperature (K)	290
Crystallization	*Bs*FolM (19 mg ml^−1^) incubated with 4 m*M* NADPH, mixed 1:1 with MCSG1 condition A1 [20%(*w*/*v*) PEG 8000, 100 m*M* HEPES pH 7.5]
Composition of reservoir solution	20%(*w*/*v*) PEG 8000, 100 m*M* HEPES pH 7.5
Volume and ratio of drop	0.4 µl:0.4 µl
Volume of reservoir (µl)	80

**Table 4:** Crystallization of FolM alternative dihydrofolate reductase 1 from *B. canis* (*Bc*FolM)

Method	Vapor diffusion, sitting drop
Plate type	Rigaku Reagents XJR
Temperature (K)	290
Crystallization	*Bc*FolM (32.3 mg ml^−1^) incubated with 6 m*M* NADPH, mixed 1:1 with 20%(*w*/*v*) PEG 8000, 100 m*M* HEPES pH 7.5
Composition of reservoir solution	20%(*w*/*v*) PEG 8000, 100 m*M* HEPES pH 7.5
Volume and ratio of drop	0.4 µl:0.4 µl
Volume of reservoir (µl)	80

**Table 5:** Data-collection and processing statistics for FolM alternative dihydro­folate reductase 1 from *B. suis* (PDB entry 5tgd, *Bs*FolM) and *B. canis* (PDB entry 5bt9, *Bc*FolM)

PDB code	5tgd	5bt9
Diffraction source	APS beamline 21-ID-F	APS beamline 21-ID-F
Wavelength (Å)	0.97872	0.97872
Temperature (K)	100	100
Detector	RayoniX MX-300 CCD	MAR Mosaic 225 mm CCD
Crystal-to-detector distance (mm)	220	130
Rotation range per image (°)	1	1
Total rotation range (°)	200	220
Space group	*P*2_1_	*P*2_1_
*a*, *b*, *c* (Å)	76.35, 76.52, 98.26	76.57, 75.60, 99.18
α, β, γ (°)	90, 109.47, 90	90, 109.23, 90
Mosaicity (°)	0.180	0.168
Resolution range (Å)	50–1.70 (1.74–1.70)	50.0–1.50 (1.54–1.50)
Total No. of reflections	491527 (36146)	783894 (57336)
No. of unique reflections	116233 (8502)	164992 (11908)
Completeness (%)	99.0 (98.4)	96.4 (94.6)
Multiplicity	4.22 (4.25)	4.8 (4.8)
〈*I*/σ(*I*)〉	19.84 (2.87)	18.26 (3.39)
*R*_r.i.m._ †	0.050 (0.486)	0.053 (0.553)
Overall *B* factor from Wilson plot (Å^2^)	18.85	15.63

† Estimated *R*_r.i.m._ = *R*_merge_[*N*/(*N* − 1)]^1/2^, where *N* is the data multiplicity.

**Table 6:** Structure-solution and refinement of FolM alternative dihydrofolate reductase 1 from *B. suis* (PDB entry 5tgd) and *B. canis* (PDB entry 5bt9)

PDB code	5tgd	5bt9
Resolution range (Å)	50–1.70 (1.74–1.70)	36.15–1.50 (1.51–1.50)
Completeness (%)	99.1	96.2
σ Cutoff	*F* > 1.34σ(*F*)	*F* > 1.35σ(*F*)
No. of reflections, working set	116170 (8678)	156072 (4573)
No. of reflections, test set	1785 (153)	8084 (235)
Final *R*_cryst_	0.163 (0.2816)	0.169 (0.2486)
Final *R*_free_	0.198 (0.2825)	0.188 (0.2877)
No. of non-H atoms		
Protein	7503	7542
Ligand	214	192
Solvent	748	724
Total	8465	8460
R.m.s. deviations		
Bonds (Å)	0.006	0.006
Angles (°)	0.828	1.132
Average *B* factors (Å^2^)		
Protein	31.9	27.7
Ligand	33.5	26.6
Water	40.0	35.1
Ramachandran plot		
Most favored (%)	96	95
Allowed (%)	4	5
